# *Azolla pinnata*, *Aspergillus terreus* and *Eisenia fetida* for enhancing agronomic value of paddy straw

**DOI:** 10.1038/s41598-018-37880-1

**Published:** 2019-02-04

**Authors:** Manveen Arora, Arvinder Kaur

**Affiliations:** Department of Zoology, Guru Nanak Dev University, Punjab, 143005 India

## Abstract

In the present study rice straw (R, control) was mixed with Cowdung (C), Azolla (A) and cellulolytic fungus *Aspergillus terreus* (F) in different combinations viz. RC, RA, RF, RCF, RCA, RFA and RCFA and subjected to aerobic composting (Acom) and vermicomposting (Vcom - with *Eisenia fetida*). It was found that addition of azolla and cattledung to two parts straw(RCA-666: 314:20 g) caused fastest degradation (105 days), gave maximum population buildup of *E*. *fetida* (cocoons, hatchlings and worm biomass), highest decline in pH, EC, TOC and C/N ratio and maximum increase over control in N(17.72%), P(44.64%), K(43.17%), H (7.93%), S (14.85%), Ca(10.16%), Na(145.97%), Fe(68.56%), Zn(12.10%) and Cu(32.24%). Rice straw (R) took longest time for degradation i.e. 120 and 140 days and had lowest content of nutrients in Vcom as well as Acom group. RCFA was also converted into Vcom at the same time but other parameters were less than RCA except for highest content of B (19.87%), Mg(21.27%) and Mn (5.58%). Bioconversion of three parts straw (RCA-735:245:20 g) was also faster (110 days) with vermicomposting than all the mixtures of Acom group (130–140 days) but nutrient content was slightly less than RCA with 2 parts straw. The results show that azolla reduces dependence on cattledung for recycling the carbon rich rice straw and enhances its agronomic value.

## Introduction

Rice (*Oryza sativa*) is one of the most widely grown crops in the world and there will be further increase in its demand as the world’s population continues to increase^1^. It is being cultivated on 166 million hectare area in the world, producing nearly 745.5 million tons (mt) of rice^2^ and 2,000 mt of rice straw^3^ annually. In India about 96 mt of rice and approximately 250 mt of straw are produced from 43 million hectares under rice cultivation^3^. This accounts for 50% share of rice straw out of the total 500 mt of crop residues^4^ produced in the country. Punjab, the Food Bowl of India, produces 10 mt of rice and 17 mt of rice straw^3^. According to an estimate around 6 tons of straw are left in the field after harvest of 4 tons of rice by mechanical harvesters^5^. This huge quantity of paddy straw is not being used economically^6^ because it is a poor feed for the cattle and resists decomposition when retained in the fields due to high silica (9–14%) and ash (22%) content^7^. High cost of collection, lack of economically viable options to utilize the straw and a short gap between harvest of rice and sowing of wheat compel the farmers to burn it in the fields^8^. According to a report of the government of Punjab^3^, around 15 mt of paddy straw was burnt in the state during the year 2014. Plumes of smoke filled with toxic emissions can be seen arising from the fields of Punjab during Oct and Nov every year^9^.

Burning of straw not only affects fertility of soil but also accounts for air and surface water pollution. It leads to emission of large amounts of suspended particulate matter and silica besides gases like CH_4_, CO, volatile organic compounds (VOC), nitrogen oxides and halogen compounds^10,11^. Heat generated by burning of straw penetrates into the soil and leads to loss of moisture, organic matter and biodiversity^8^. It has been estimated that burning of one ton of straw accounts for releasing 3 kg of particulate matter, 60 kg of CO, 1,460 kg of CO_2_, 199 kg of ash and 2 kg of SO_2_^12^ along with a loss of 5.5 kg nitrogen, 2.3 kg phosphorus, 1.2 kg sulfur and a huge quantity of organic carbon. Therefore there is a strong need to recycle this organic resource in a way that it not only yields plant nutrients but also protects/conserves physico- chemical characteristics of air, water and soil.

In the present study the rice straw was spiked with *Aspergillus terreus* (F), *Azolla pinnata* (A) & cattledung (C) and subjected to vermicomposting (Vcom with *Eisenia fetida*) and aerobic composting (Acom) for its conversion into a value added product. Aerobic composting is a time immemorial process but Vermicomposting (a biodegradation process using microbes and earthworm) is gaining popularity for biotransformation of organic solid wastes into fertilizer^13,14^. *A*. *terreus* is a cellulolytic fungus that produces enzymes like cellulases, hemicellulases and ligninases^15^ it was expected that it will enhance bacterial action after initial breakdown of complex carbohydrates during composting and vermicomposting. Earlier Suthar^16^ reported enhancement of degradation of cellulose rich industrial sludge when it was spiked with brown rot fungus (*Oligosporus placenta)* and subjected to vermicomposting. Azolla a free floating aquatic weed^17^ that lives in association with a blue-green alga, *Anabaena azollae* (that fixes atmospheric nitrogen) was used as a nitrogen supplement and source of nutrients. The weed biomass rich in fibres, proteins, lipids, carbohydrates, minerals and vitamins can be efficiently used for vermicomposting nitrogen deficient organic wastes^18,19^. It is found abundantly in waste water ponds in the villages. Cattle dung was used as a microbial inoculum as it contains bacteria, protozoa and yeast (a bioresource available in huge quantities but mostly washed off in water^20^). Different combinations were evaluated for the rate of bioconversion and for quality of the product from this crop residue. This study is first of its kind to use combination of fungus, azolla and cattledung for enhancing the bioconversion of rice straw into a nutrient rich plant growth medium.

## Results

### Degradation time

Degradation of the mixtures of Vcom group was observed after 105 to 120 days (90–105 days post earthworm inoculation) while in Acom group it occurred after 130–140 days (Table 1). The time required for bioconversion was different for various mixtures but it was much less for the Vcom group in comparison to the Acom group. Minimum time was required for bioconversion of both RCFA and RCA (total 105 days and 130 days) in the Vcom and Acom groups respectively. Post pre-composting, 98 days (total 113 days) were required for RCF and RFA while 100, 102, 103 and 105 days were required for RA, RC, RF and R respectively in the Vcom group. In the Acom group RFA, RCF, RA, RC and RF were ready in 135 days. Maximum time of degradation was observed for R in both the groups (total 120 in the Vcom group and 140 days in the Acom group).

**Table 1:** Composition per kg and time of bioconversion of various mixtures of rice straw, cattledung, azolla and fungus.

Materials used	Treatments	Vcom group (days)	Acom group (days)
Rice straw (1000 g)	R	120	140
Rice straw (666 g) + Cattle Dung (334 g)	RC	117	135
Rice straw (980 g) + Azolla (20 g)	RA	115	135
Rice straw (980 g) + Fungus (20 ml)	RF	118	135
Rice straw (666 g) + Cattle dung (314 g) + Fungus (20 ml)	RCF	113	135
Rice straw (666 g) + Cattle dung (314 g) + Azolla (20 g)	RCA	105	130
Rice straw (960 g) + Fungus (20 ml) + Azolla(20 g)	RFA	113	135
Rice straw (666 g) + Cattle dung (294 g) + Azolla(20 g) + Fungus (20 ml)	RCFA	105	130

### Population buildup of earthworms

**Figure 1:**
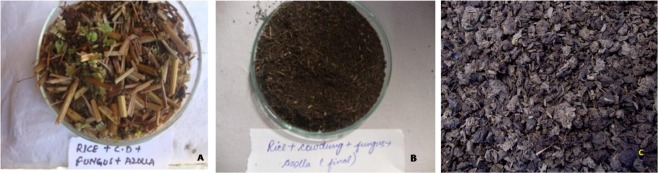
Appearance of the initial mixture RCFA (**A**) and its products (**B**) Vcom, (**C**) Acom.

A significant difference (p < 0.01) was observed in the worm biomass and numbers of cocoons, hatchlings and worms in various mixtures (Fig. 1). Number of earthworms increased till 80^th^ day after inoculation and then declined in all the mixtures. The number of worms in RCA was (225.67 ± 3.84) > RCFA (209.67 ± 0.88) > RC (193.34 ± 0.88) > RCF (159.67 ± 0.88) > RFA (157.34 ± 1.20) > RA (148.00 ± 1.52) > RF (147.34 ± 1.20) > R (132.34 ± 0.88) on 80^th^ day. On 105^th^ day also, same trend was observed for various treatments. Weight of earthworms showed a similar trend and declined with passage of time. Earthworms showed rapid growth in all the treatments till 80 days, after that a decline continued till termination. On 80^th^ day, the biomass was maximum in RCA (87.47 ± 0.75) and minimum in R (62.34 ± 0.66) and the trend continued till the end of the experiment (105^th^ day) (Fig. 1A). Production of cocoons started in the third to fourth week in different treatments and increased till 80^th^ day but a decline was observed thereafter. Maximum number of cocoons was observed in RCFA (434.33 ± 1.20) which was followed by RCA (406.33 ± 3.18) on 80^th^ day while on 105^th^ day RCA had maximum number of cocoons (204 ± 3.92) and R had minimum number (91.67 ± 1.45). Maximum number of hatchlings was seen in RCFA (340.67 ± 4.67) followed by RCA (309.00 ± 2.08) while it was minimum in R (155.6 ± 6.93).

### Physico-chemical composition

Tables 3, 4 and 5 show the initial and final physico-chemical composition of the mixtures. The rice straw was taken as control and other treatments were compared separately with the control of each group. Percent change over initial was calculated for rice straw for observing the change in various parameters of the product. In both the groups an increase over initial (p < 0.01) was observed for N, P, K, Na, Cu, B, Mn, Zn, Mg, Ca, Fe whereas a decrease over initial was observed for TOC, C/N, pH, and EC. In the Vcom group, the increase for N (81.95%), P (47.70%), K (26.08%), Na (169.23%), Ca (105.97%), Mg (66.67%), B (82.63%), Cu (31.04%), Mn (81.62%), Zn(23.07%), Fe (25.30%), H (5.71%) and S (86.66%) as well as the decrease for TOC (28.29%), C/N(60.58%), pH (10.03%), and EC (27.97%) was more than the Acom group. In the Acom group, the increase for N, P, K, Na, Mg, Ca, Cu, B, Mn, Zn, Fe, H and S was 61.58%, 13.71%, 11.87%, 138.11%, 51.09%, 19.46%, 48.92%, 4.16%, 52.93%, 69.01%, 9.85%, 1.60% and 71.95% respectively whereas the decrease for TOC C/N, pH, and EC was 26.86%, 54.74%, 7.82%, 9.87% respectively. A significantly higher increase over initial (p < 0.01) was observed in all the physico-chemical characteristics of the mixtures in the Vcom group compared to the Acom group.

**Table 2:** Physico-chemical characteristics of Rice straw, Cattledung and Azolla (Mean ± SE).

Parameters	Rice Straw	Cattledung	Azolla
pH	8.63 ± 0.01	7.78 ± 0.05	7.82 ± 0.02
EC (µS/cm)	0.91 ± 0.01	1.06 ± 0.02	1.54 ± 0.03
TOC (g/kg)	385.33 ± 1.38	323.33 ± 1.76	300 ± 2.31
Nitrogen (g/kg)	9.18 ± 0.05	13.20 ± 0.13	18.56 ± 0.19
C:N	41.99 ± 0.20	24.50 ± 0.38	16.16 ± 0.25
Phosphorus (g/kg)	2.10 ± 0.02	5.20 ± 0.11	4.23 ± 0.06
Potassium (g/kg)	16.51 ± 0.09	11.36 ± 0.27	15.31 ± 0.17
Sodium (g/kg)	0.48 ± 0.02	1.53 ± 0.02	3.46 ± 0.05
Calcium (g/kg)	6.59 ± 0.03	6.18 ± 0.04	9.14 ± 0.05
Magnesium (g/kg)	4.02 ± 0.02	4.87 ± 0.05	5.77 ± 0.04
Iron (mg/kg)	1237.33 ± 9.16	1979 ± 7.811	4542.33 ± 9.84
Zinc (mg/kg)	106.21 ± 0.15	164.62 ± 1.21	144.50 ± 0.94
Boron (mg/kg)	16.58 ± 0.13	7.57 ± 0.04	8.78 ± 0.11
Copper (mg/kg)	10.40 ± 0.06	15.08 ± 0.05	8.58 ± 0.23
Manganese (mg/kg)	211.64 ± 0.43	114.53 ± 0.47	321.78 ± 1.64
Sulphur	2.06 ± 0.01	3.08 ± 0.02	3.36 ± 0.05
Hydrogen	49.31 ± 0.09	49.15 ± 0.13	32.76 ± 0.19

**Table 3:** Initial physico-chemical characteristics of various mixtures of rice straw, cattledung, azolla and fungus (Mean ± SE).

Treatments	R	RC	RA	RF	RCF	RCA	RFA	RCFA
Parameters								
pH	8.63 ± 0.01	8.53 ± 0.01	8.43 ± 0.01	8.57 ± 0.01	8.42 ± 0.01	8.23 ± 0.01	8.40 ± 0.01	8.32 ± 0.01
EC (µS/cm)	0.91 ± 0.01	0.86 ± 0.01	0.85 ± 0.01	0.88 ± 0.01	0.74 ± 0.01	0.74 ± 0.01	0.78 ± 0.01	0.77 ± 0.01
C (g/kg)	385.33 ± 1.38	376.58 ± 1.07	365.67 ± 1.38	378.00 ± 2.03	374 ± 4.93	360.17 ± 1.28	365.33 ± 1.61	362.67 ± 1.33
N (g/kg)	9.18 ± 0.05	9.51 ± 0.06	9.84 ± 0.04	9.30 ± 0.04	9.38±0.53	11.17 ± 0.04	9.41 ± 0.06	11.04 ± 0.09
C/N	41.99 ± 0.20	39.61 ± 0.23	37.18 ± 0.25	40.66 ± 0.18	39.89 ± 0.38	32.26 ± 0.16	38.82 ± 0.25	32.87 ± 0.32
P (g/kg)	2.10 ± 0.02	2.24 ± 0.04	2.43 ± 0.02	2.17 ± 0.01	2.48 ± 0.01	3.01 ± 0.05	2.86 ± 0.01	2.65 ± 0.01
K (g/kg)	16.51 ± 0.09	18.57 ± 0.07	20.53 ± 0.04	17.76 ± 0.07	19.10 ± 0.06	22.92 ± 0.03	21.25 ± 0.06	22.30 ± 0.08
Na (g/kg)	0.48 ± 0.02	0.65 ± 0.02	0.73 ± 0.01	0.58 ± 0.01	0.73 ± 0.01	0.99 ± 0.01	0.77 ± 0.01	0.92 ± 0.01
Ca (g/kg)	6.59 ± 0.03	6.85 ± 0.02	7.14 ± 0.02	6.75 ± 0.01	7.22 ± 0.01	7.85 ± 0.02	7.60 ± 0.05	7.77 ± 0.02
Mg (g/kg)	4.02 ± 0.02	4.89 ± 0.03	4.38 ± 0.01	4.18 ± 0.04	4.63 ± 0.02	5.48 ± 0.03	4.14 ± 0.04	5.90 ± 0.03
Fe (mg/kg)	1237.33 ± 9.16	1369.67 ± 5.52	1468.00 ± 6.07	1134.33 ± 6.73	1554.17 ± 10.89	1933 ± 9.82	1647 ± 7.55	1873.50 ± 10.62
Zn (mg/kg)	106.21±0.15	113.43 ± 0.06	118.50 ± 0.15	108.49 ± 0.08	110.44 ± 0.07	126 ± 0.21	120.58 ± 0.10	125.54 ± 0.11
Cu (mg/kg)	10.40 ± 0.06	13.40 ± 0.05	14.53 ± 0.11	13.18 ± 0.05	13.86 ± 0.03	15.93 ± 0.01	13.32 ± 0.08	15.22 ± 0.21
B (mg/kg)	16.58 ± 0.13	18.04 ± 0.04	18.50 ± 0.13	17.19 ± 0.03	19.24 ± 0.04	22.09 ± 0.03	21.37 ± 0.10	22.63 ± 0.07
Mn (mg/kg)	211.65 ± 0.43	221.39 ± 0.27	230.93 ± 0.24	216.15 ± 0.22	225.18 ± 0.23	236.22 ± 0.26	232.63 ± 0.27	238.01 ± 0.27
H (g/kg)	49.31 ± 0.09	50.46 ± 0.15	51.27±0.04	50.03 ± ± 0.05	51.76 ± 0.16	52.97 ± 0.06	52.00 ± 0.03	52.40 ± 0.11
S (g/kg)	2.06 ± 0.02	2.28 ± 0.03	2.38 ± 0.02	2.19 ± 0.02	2.23 ± 0.07	2.85 ± 0.01	2.53 ± 0.02	2.65 ± 0.02

**Table 4:** Physico-chemical characteristics of the products of various mixtures of rice straw, cattledung, azolla and fungus (Mean ± SE).

Parameters	Treatments	R	RC	RA	RF	RCF	RCA	RFA	RCFA
pH	Vcom	7.76 ± 0.01^a^	7.63±0.01^c^ (−1.72)	7.58 ± 0.01^d^ (−2.34)	7.69 ± 0.02^b^ (−0.99)	7.48 ± 0.01^d^ (−3.71)	7.27 ± 0.02 ^f^ (−6.35)	7.56 ± 0.01^d^ (−2.64)	7.38 ± 0.01^e^ (−4.94)
	Acom	7.95 ± 0.01^a^	7.85 ± 0.01^bc^ (−1.34)	7.81 ± 0.01^c^ (−1.82)	7.89 ± 0.01^b^ (−0.84)	7.68 ± 0.02^e^ (−3.42)	7.47 ± 0.01 ^g^ (−6.08)	7.76 ± 0.01^d^ (−2.49)	7.57 ± 0.02 ^f^ (−4.78)
EC (µS/cm)	Vcom	0.66 ± 0.01^a^	0.62 ± 0.01^abc^ (−5.84)	0.61 ± 0.01^abc^ (−7.11)	0.63 ± 0.01^ab^ (−4.06)	0.58 ± 0.01 ^cd^ (−12.18)	0.55 ± 0.01^d^ (−16.24)	0.59 ± 0.01^bcd^ (−10.66)	0.57 ± 0.01 ^cd^ (−12.94)
	Acom	0.82 ± 0.01^a^	0.77 ± 0.1^b^ (−6.49)	0.77 ± ± 0.01^b^ (−6.69)	0.79 ± 0.01^ab^ (−4.26)	0.69 ± 0.05 ^cd^ (−15.82)	0.63 ± 0.01^e^ (−23.33)	0.71 ± 0.01^c^ (−14.20)	0.65 ± 0.01^de^ (−21.10)
C (g/kg)	Vcom	276.33 ± 0.67^a^	263.67 ± 1.52^b^ (−4.58)*	256.50 ± 2.59^bc^ (−7.18)	263.33 ± 2.43^b^ (−4.70)	261.83 ± 1.25^bc^ (−5.25)	254.17 ± 1.14^c^ (8.02)	258.67 ± 1.26^bc^ (−6.51)	260.83 ± 1.82^bc^ (−5.61)
	Acom	281.83 ± 0.79^a^	275.17 ± 1.83^b^ (−2.37)	265.67 ± 1.05^cde^ (−5.74)	269.33 ± 0.71^bcd^ (−4.44)	270.67 ± 0.95^bc^ (−3.96)	260.17 ± 1.82^e^ (−7.69)	261.00±2.08^e^ (−7.48)	263.17 ± 1.11^de^ (−6.62)
N (g/kg)	Vcom	16.70 ± 0.10^f^	17.27 ± 0.12^de^ (3.41)	17.57 ± 0.05 ^cd^ (5.19)	16.91 ± 0.03^ef^ (1.26)	17.8C9 ± 0.03^c^ (7.14)	19.66 ± 0.14^a^ (17.72)	17.95 ± 0.07^c^ (7.48)	18.70 ± 0.12^b^ (11.98)
	Acom	14.83 ± 0.03^c^	15.15 ± 0.08^c^ (2.16)	15.57 ± 0.11^b^ (4.97)	14.79 ± 0.07^c^ (0.28)	15.84 ± 0.04^ab^ (6.83)	15.94 ± 0.14^a^ (7.51)	15.71 ± 0.04^ab^ (5.9)	15.95 ± 0.02^a^ (7.55)
C/N	Vcom	16.55 ± 0.11^a^	15.27 ± 0.10^b^ (−7.74)	14.60 ± ± 0.15^c^ (−11.77)	15.57 ± 0.15^b^ (−5.90)	14.63 ± 0.09^c^ (−11.57)	12.93 ± 0.12^e^ (−21.86)	14.39 ± 0.11 ^cd^ (−13.02)	13.95 ± 0.17^d^ (−15.69)
	Acom	19.01 ± 0.09^a^	18.16 ± 0.15^b^ (−4.42)	17.07 ± 0.09^c^ (−10.19)	18.22 ± 0.14^b^ (−4.15)	17.08 ± 0.05^c^ (−10.11)	16.32 ± 0.17^e^ (−14.11)	16.62 ± 0.12 ^cd^ (−12.56)	16.50 ± 0.07^d^ (−13.18)
P (g/kg)	Vcom	3.11 ± 0.01^g^	3.61 ± 0.04^d^ (16.04)	3.48 ± 0.02^e^ (12.12)	3.29 ± 0.02 ^f^ (5.90)	3.75 ± 0.02^c^ (20.83)	4.49 ± 0.02^a^ (44.64)	3.66 ± 0.05 ^cd^ (17.65)	3.88 ± 0.02^b^ (24.89)
	Acom	2.39 ± 0.04^f^	2.67 ± 0.02^d^ (11.57)	2.57 ± 0.02^e^ (7.39)	2.46 ± 0.01 ^f^ (2.72)	2.73 ± 0.02 ^cd^ (14.01)	3.24 ± 0.04^a^ (35.4)	2.77 ± 0.03^c^ (15.96)	2.95 ± 0.02^b^ (23.28)
K (g/kg)	Vcom	20.81 ± 0.25^f^	24.05 ± 0.34^d^ (15.57)	27.73 ± 0.07^b^ (33.22)	22.42 ± 0.07^e^ (7.7)	25.14 ± 0.35^c^ (20.76)	29.80 ± 0.16^a^ (43.17)	27.56 ± 0.14^b^ (32.42)	28.24 ± 0.05^b^ (35.68)
	Acom	18.47 ± 0.08^e^	21.22 ± 0.02^c^ (14.89)	24.08 ± 0.53^b^ (30.39)	19.64 ± 0.06^d^ (6.34)	21.66 ± 0.06^c^ (17.28)	26.32 ± 0.13^a^ (42.5)	24.43 ± 0.16^b^ (32.25)	24.98 ± 0.06^b^ (35.25)
Na (g/kg)	Vcom	1.28 ± 0.01^f^	2.76 ± 0.04^d^ (114.94)	2.42 ± 0.01^c^ (88.7)	1.69 ± 0.10^e^ (31.3)	2.49 ± 0.01^c^ (94.03)	3.16 ± 0.03^a^ (145.97)	2.58 ± 0.03^c^ (101.3)	2.82 ± 0.04^b^ (119.61)
	Acom	1.14 ± 0.02^g^	2.28 ± 0.02^bc^ (101.17)	2.09 ± 0.01^e^ (84.14)	1.45 ± 0.04 ^f^ (28.05)	2.18 ± 0.02^d^ (92.36)	2.43 ± 0.02^a^ (113.66)	2.25 ± 0.41 ^cd^ (98.24)	2.36 ± 0.02^ab^ (107.78)
Ca (g/kg)	Vcom	13.58 ± 0.02^h^	13.91 ± 0.02 ^f^ (2.41)	14.12 ± 0.02^e^ (3.96)	13.75 ± 0.02 ^g^ (1.24)	14.30 ± 0.01^d^ (5.33)	14.96 ± 0.02^a^ (10.16)	14.60 ± 0.02^c^ 7.51	14.78 ± 0.02^b^ (8.86)
	Acom	11.14 ± 0.01^h^	11.39 ± 0.03 ^f^ (2.23)	11.50 ± 0.01^e^ (3.16)	11.25 ± 0.01 ^g^ (0.99)	11.71 ± 0.03^d^ (5.06)	12.26 ± 0.01^a^ (9.98)	11.97 ± 0.02^c^ (7.16)	12.05 ± 0.02^b^ (8.14)
Mg (g/kg)	Vcom	6.69 ± 0.02^g^	7.36 ± 0.02^d^ (9.99)*	7.13 ± 0.05^e^ (6.6)	6.87 ± 0.05 ^f^ (2.64)	7.65 ± 0.01^c^ (14.27)	7.93 ± 0.02^b^ (18.46)	7.82 ± 0.01^b^ (16.81)	8.12 ± 0.02^a^ (21.27)
	Acom	6.14 ± 0.02^d^	6.59 ± 0.13^c^ (7.27)	6.30 ± 0.08^d^ (2.55)	6.30 ± 0.06^d^ (2.52)	6.82 ± 0.06^bc^ (11.13)	7.22 ± 0.32^a^ (17.59)	7.10 ± 0.03^ab^ (15.58)	7.37 ± 0.02^a^ (19.98)
Fe (mg/kg)	Vcom	1550.33 ± 6.49^g^	1655.67 ± 9.11 ^f^ (6.79)	1957.17 ± 7.10^d^ (26.24)	1587 ± 4.02 ^g^ (2.37)	2131.50 ± 6.43^c^ (37.49)	2487.50 ± 13.74^a^ (60.45)	1859.17 ± 5.36^e^ (19.92)	2353.83 ± 12.26^b^ (51.83)
	Acom	1359.17 ± 8.20^f^	1463.33 ± 5.64^a^ (7.66)	1759.33 ± 4.87^d^ (29.44)	1423.67 ± 4.34^e^ (4.75)	1878.17 ± 8.40^c^ (38.19)	2291 ± 14.0^a^ (68.56)	1778.50±5.70^d^ (30.85)	2085.67 ± ± 18.27^b^ (53.45)
Zn (mg/kg)	Vcom	130.71 ± 0.20^g^	136.26 ± 0.16^a^ (4.24)	139.35 ± 0.15^d^ (6.6)	132.76 ± 0.19 ^f^ (1.57)	135.48 ± 0.11^e^ (3.65)	146.53 ± 0.10^a^ (12.1)	142.13 ± 0.14^c^ (8.73)	145.06 ± 0.31^b^ (10.97)
	Acom	110.63 ± 0.17^g^	119.85 ± 0.19^d^ (8.34)	121.87 ± 0.24^c^ (10.16)	115.15 ± 0.10 ^f^ (4.08)	117.33 ± 0.19^e^ (6.05)	130.73 ± 0.28^a^ (18.17)	126.07 ± 0.23^b^ (13.96)	129.78 ± 0.12^a^ (17.39)
Cu (mg/kg)	Vcom	13.63 ± 0.04^f^	15.62 ± 0.09^a^ (14.63)	16.56 ± 0.09^c^ (21.52)	15.48 ± 0.10^e^ (13.6)	17.46 ± 0.08^d^ (28.08)	18.34 ± 0.01^a^ (34.55)	17.39 ± 0.16^e^ (27.60)	18.21 ± 0.01^b^ (33.58)
	Acom	12.42 ± 0.14^f^	14.14 ± 0.04^a^ (13.78)	14.97 ± 0.04^d^ (20.5)	13.95 ± 0.03^e^ (12.29)	15.90 ± 0.04^bc^ (27.96)	16.43 ± 0.14^a^ (32.24)	15.70 ± 0.0^c^ (26.39)	16.06 ± 0.05^b^ (29.27)
B (mg/kg)	Vcom	30.28 ± 0.08^g^	33.16 ± 0.10^a^ (9.5)	34.85 ± 0.04^c^ (15.09)	32.19±0.10 ^f^ (6.29)	34.56 ± 0.10 ^cd^ (14.12)	35.59 ± 0.26^b^ (17.52)	34.43 ± 0.08^d^ (13.70)	36.30 ± 0.10^a^ (19.87)
	Acom	25.05 ± 0.14^d^	25.74 ± 0.11 ^cd^ (2.75)	26.15±0.08^c^ (4.38)	25.32 ± 0.14^d^ (1.04)	28.28 ± ± 0.38^b^ (12.89)	29.00 ± 0.05^b^ (15.73)	28.43 ± 0.19^b^ (13.46)	29.92 ± 0.12^a^ (19.44)
Mn (mg/kg)	Vcom	384.42±1.10 ^h^	392.48 ± 0.16 ^f^ (2.1)	398.54 ± 0.14^d^ (3.68)	390.06 ± 0.18 ^g^ (1.47)	394.55 ± 0.17^e^ (2.64)	403.41 ± 0.22^b^ (4.94)	401.06 ± 0.26^c^ (4.33)	405.88 ± 0.21^a^ (5.58)
	Acom	315.20 ± 0.35^h^	319.32 ± 0.19 ^f^ (1.3)	323.64 ± 0.13^d^ (2.67)	316.93 ± 0.11 ^g^ (0.55)	320.88 ± 0.24^e^ (1.8)	327.33 ± 0.23^b^ (3.85)	325.91 ± 0.17^c^ (3.40)	329.71 ± 0.15^a^ (4.6)
H(g/kg)	Vcom	52.13 ± 0.06^g^	53.27 ± 0.0^e^ (2.20)	54.26 ± 0.05^d^ (4.10)	52.94 ± 0.04 ^f^ (1.55)	53.95 ± 0.07^d^ (3.49)	56.26 ± 0.11^a^ (7.93)	54.88 ± 0.04^c^ (5.29)	55.37 ± 0.08^b^ (6.22)
	Acom	50.10 ± 0.05^f^	51.19 ± 0.07^e^ (2.17)	52.13 ± 0.05 ^cd^ (4.06)	50.80 ± 0.05^e^ (1.40)	51.68 ± 0.06^d^ (3.14)	53.40 ± 0.15^a^ (6.58)	52.17 ± 0.07^bc^ (4.13)	52.58 ± 0.07^b^ (4.95)
S (g/kg)	Vcom	3.85 ± 0.04^d^	4.12 ± 0.03^c^ (7.17)	4.27 ± 0.01^bc^ (10.96)	3.94 ± 0.02^d^ (2.34)	4.16 ± 0.02^bc^ (7.97)	4.42 ± 0.05^a^ (14.85)	4.26 ± 0.02^bc^ (10.74)	4.30 ± 0.04^ab^ (11.61)
	Acom	3.55 ± 0.03^c^	3.79 ± 0.05^b^ (6.96)	3.88 ± 0.05^b^ (9.40)	3.62 ± 0.02^c^ (2.07)	3.82 ± 0.01^b^ (7.80)	4.04 ± 0.02^a^ (13.82)	3.89 ± 0.02^ab^ (9.78)	3.94 ± 0.03^ab^ (11)

Values with different superscripts are significant at (p < 0.01).

Values in parenthesis are percent change over control.

**Table 5:** Physico-chemical characteristics (Mean ± SE) of initial and final 3:1:20 RCA (735 g + 245 g + 20 g).

Treatments	RCA (Initial)	RCA(Vcom)	RCA(Acom)
Parameters			
C (g/kg)	365.50 ± 1.52	260.54 ± 0.83	265.67 ± 0.76
N (g/kg)	11.05 ± 0.02	19.09 ± 0.05	15.23 ± 0.08
C/N	33.07 ± 0.15	13.65 ± 0.07	17.44 ± 0.12
P (g/kg)	3.30 ± 0.02	4.26 ± 0.3	3.59 ± 0.03
K (g/kg)	23.02 ± 0.02	30.11 ± 0.04	26.78 ± 0.07
Na (g/kg)	0.89 ± 0.01	3.07 ± 0.03	2.12 ± 0.03
Ca (g/kg)	7.76 ± 0.01	14.88 ± 0.03	12.05 ± 0.02
Mg (g/kg)	5.24 ± 0.03	7.68 ± 0.04	7.05 ± 0.02
Fe (mg/kg)	1904.17 ± 4.49	2442 ± 6.49	2224.50 ± 8.12
Zn (mg/kg)	129.26 ± 0.47	148.38 ± 0.43	132.67 ± 0.37
Cu (mg/kg)	15.35 ± 0.07	18.04 ± 0.05	15.90 ± 0.05
B (mg/kg)	21.59 ± 0.13	34.54 ± 0.13	28.33 ± 0.10
Mn (mg/kg)	233.06 ± 0.62	401.20 ± 0.32	324.83 ± 0.69
pH	8.20 ± 0.01	7.24 ± 0.01	7.43 ± 0.01
EC	0.77 ± 0.01	0.57 ± 0.01	0.66 ± 0.01
H (mg/kg)	52.01 ± 0.06	55.99 ± 0.04	53.21 ± 0.07
S (mg/kg)	2.44 ± 0.07	4.01 ± 0.11	3.95 ± 0.08

A significant decrease over control (p < 0.01) was observed in the content of total organic carbon (TOC), C/N ratio, pH and EC while a significant increase over control was observed for N, P, K, H, S, Na, Ca, Mg, Cu, B, Fe, Zn and Mn for all the mixtures of Vcom as well as Acom group. However, decrease and increase in the parameters of the Vcom group were significantly more (p < 0.01) than the Acom group.

### Macronutrients

Maximum reduction over control in TOC was observed for RCA (−8.02%) followed by RA (−7.18%) while it was minimum in RC (−4.58%) in the Vcom group. Decline over control in the total organic carbon content ranged from −2.37 to −7.69% in various mixtures of Acom group. In the Vcom group 17.72% (maximum) increase over control for nitrogen was observed in RCA while it was only 1.26% (minimum) in RF. In the Acom group increase over control was minimum in RF (0.28%) but maximum in RCFA (7.55%). The initial as well as final C/N ratio of both Acom and Vcom groups was minimum in RCA and maximum in R. The initial range of C/N ratio of the mixtures was 32.26–41.99 whereas it came down to 12.93–16.55 and 16.32–19.01 in the Vcom and Acom group respectively. The reduction over control in the C/N ratio of Vcom and Acom mixtures was maximum in RCA (−21.86% and −14.11%) and minimum in RF (−5.90% and −4.15%) of the Vcom and Acom groups respectively.

Maximum increase over control in P was observed in RCA (44.64%) while it was minimum in RF(5.90%) of the Vcom group, however, increase over control in P of the Acom group ranged from 35.40%(RCA)–2.72% (RF) only.

Increase over control for K in the Vcom group was 43.17% in RCA, 35.68% in RCFA, 33.22% in RA, 32.42% in RFA, 20.76% in RCF, 15.57% in RC and 7.70% in RF. In the Acom group on the other hand increase in the content of K was less than the Vcom group and ranged from 6.34% (RF) to 42.50% (RCA).

The increase over control for H ranged from 1.15–7.93% in Vcom and 1.40–6.585 in Acom for RF and RCA respectively. The increase in sulphur (S) over control was maximum in RCA (14.85%) and minimum in RF (2.34%) for the Vcom group and in the Acom group maximum increase occurred in RCA (13.82%) while minimum in RF (2.07%). The initial pH of R, C and A was 8.63, 7.78 and 7.80 and out of all the mixtures it was minimum in the mixture RCA (8.23). The trend of decline over control was RCA (−6.35% and −6.08%) > RCFA (−4.94% and −4.78%) > RCF (−3.71% and −3.42%) > RFA (−2.64% and −2.49%) > RA (−2.34% and −1.82%) > RC (−1.72% and −1.34%) > RF (−0.99% and −0.84%) in the Vcom and Acom group respectively. Electrical Conductivity was more in the final mixtures of Acom group in comparison to the Vcom group. However, the percent decline over control was more in the Acom group. The trend of decrease over control was RCA (−23.33 and −16.24) > RCFA (−21.10% and −12.94%) > RCF (−15.82% and −12.19%) > RFA (−14.20% and −10.66%) > RA (−6.69% and −7.11%) > RC (−6.49% and −5.84%) > RF (−4.26% and −4.06%) > R in the Acom and Vcom group respectively.

In the Vcom group the increase over control for sodium (Na) was 31.30–145.97% and in the Acom group it was 28.05–113.66% in RF and RCA respectively. In the Vcom group increase over control for calcium (Ca) was 10.16% in RCA, 8.86% in RCFA, 7.51% in RFA, 5.33% in RCF, 3.96% in RA, 2.41% in RC and 1.24% in RF whereas in the Acom group the increase over control was in the range of 0.99% (RF)–9.98% (RCA). The increase over control for Mg ranged from 2.64–21.27% in the Vcom group and from 2.52–19.98% in the Acom group in RCFA and RF respectively.

### Micronutrients

Iron (Fe) and zinc (Zn) were more in the products of Acom group while boron (B), manganese (Mn) and copper (Cu) were more in the products of Vcom group. In the Vcom group there was 2.37% (RF)–60.45% (RCA) increase in Fe, whereas the increase over control in the Acom group ranged from 4.75% (RF)–68.56% (RCA). Increase over control in the content of Zinc was 1.57% (RF)–12.10% (RCA) in the Vcom group and 4.08% (R)–18.17% (RCA) in the Acom group. Trend of increase in the Vcom and Acom group was RCA > RCFA > RFA > RA > RC > RCF > RF > R. For Boron, the increase over control ranged from 6.29 (RF)–19.87% (RCFA) in the Vcom group while it ranged from 1.04% (RF)–19.44% (RCFA) in the Acom group. The increase over control for manganese (Mn) was maximum in RCFA (5.58% and 4.60%)  followed by RCA (4.94% and 3.85%) and it was minimum in RF (1.47% and 0.55%) for the Vcom and Acom group respectively. The increase over control in the content of copper (Cu) was 34.55% (RCA)–13.60% (RF) and 32.24% (RCA)–12.29% (RF) in the Vcom and Acom group respectively.

The mixture RCA with 3 parts straw had almost similar physico-chemical characteristics and population buildup as the mixture with 2 parts of straw. The time of degradation was more as it took 110 and 141 days for degradation of Vcom and Acom mixture respectively.

## Discussion

In the present study rate of degradation was faster for mixtures of Vcom group in contrast to Acom group. In both the groups RCFA and RCA were the first mixtures ready for harvesting. The difference in feed mixtures could be responsible for variation in the time required for degradation. Faster degradation of the mixtures in the Vcom group could have been due to combined action of earthworms and microbes. A variety of intestinal microorganisms are present in the gut of earthworm which produce digestive enzymes such as amylase, proteases, lipases and cellulases that enhance biodegradation of organic matter^21–23^.

In the present study better growth rate of earthworms in RCA can be due to more acceptability of this mixture as feed by the earthworms and/or due to the presence of components that were favorable for their growth^24^. An increase in earthworm biomass has also been reported by Suthar during vermicomposting of agricultural waste with cow dung^25^. Peak weight of earthworms on 80^th^ day and decline afterwards in the present study is similar to the results of Shak *et al*.^26^ who have reported similar trend in weight loss in *E*. *eugeniae* after 30 days of vermicomposting. They reported more growth and reproduction of earthworms in rice straw compared to rice husk. Similar trend of weight loss due to food shortage by the end of vermicomposting has been reported by Suthar^27^, Kaur *et al*.^28^ and Yadav & Garg^29^. An increase in earthworm biomass on 45^th^ day in weed *Salvinia natans* with highest proportion of cattle dung (40%) has been related to easily metabolizable organic matter and non-assimilated carbohydrates essential for growth and reproduction of earthworms^30^. In the present study RCA and RCFA had maximum number of cocoons and hatchlings which suggests more availability of nutrients in these mixtures. Improvement in the contents of nitrogen and nutrients with the addition of azolla might have provided a conducive environment for growth of microbes and *E*. *fetida*. It is expected that along with initial breakdown of straw, fungus *A*. *terreus* might have enhanced growth as well as reproduction of worms because earthworms selectively feed on fungi^31^. Increase in cocoons and hatchlings during vermicomposting of the mixture of rice straw and cow dung has been related to its better biochemical quality for the reproduction of earthworms^26,32,33^. Apart from biochemical properties, the microbial biomass and decomposition activities during vermicomposting also determine worm biomass and cocoon production^34^ which supports the observed maximum biomass in RCA in the present study.

The decline in TOC with passage of time in both Vcom and Acom groups of the present study can be attributed to microbial oxidation of the labile forms of carbon to carbon dioxide^35,36^. Higher decline in TOC in the Vcom group could be due to respiration by microbes and earthworms as well as assimilation of carbon as microbial and earthworm biomass^37^. It is clear that paddy straw spiked with azolla showed maximum decrease in TOC which can be due to higher population density of earthworms and diversity of microbes in this mixture. More increase in the content of nitrogen in the mixture could also have enhanced degradation of the straw and caused higher decline in carbon. The mutualistic effect of gut microbes and earthworms has been linked to intensified mineralization of carbon in the waste mixtures during vermicomposting process^24^, which is evident from a significantly more decline in C in all the mixtures of Vcom group. Presence of azolla with nitrogen fixing blue green algae *Anabaena azollae* seems clearly to be responsible for higher increment of nitrogen in the respective mixtures. Higher decline in pH of RCA (6.08% over control) may also be another reason for nitrogen retention and faster decomposition because excess of nitrogen is generally lost as volatile ammonia at higher pH values^38^. Bansal & Kapoor^39^ reported that final nitrogen content of the compost is dependent on the initial content of nitrogen in the waste, this supports the observed higher N content in the mixtures having azolla in both the groups of the present study. Microclimate of the mixtures has been reported to be altered by earthworms^24^ that may have promoted microbial activity responsible for nitrogen enrichment during vermicomposting in the present study.

A comparison of C/N ratio of the initial and final material reflects expected mineralization of organic waste and stabilization during composting and vermicomposting and indicates suitability of prepared vermicompost for agricultural use. Addition of azolla brought an increase in nitrogen content of the initial material thus balancing the C/N ratio for earlier degradation of the mixtures with azolla in both Vcom and Acom groups. At higher values of C/N the degradation of material is either less or delayed^37^, which supports maximum time of degradation of R in the present study. Prominent role of earthworms in bringing down C/N ratio is clearly evident from the significant difference in C: N ratio of the products of Vcom and Acom group of the present study. The wide difference in the C/N ratio of both RCA and RCFA from other treatments of both the groups could have been due to differences in palatability, physical structure and microbial diversity of various mixtures as suggested by Yadav and Garg^29^. C/N ratio depicts the change in carbon and nitrogen spectra of feed material and a reduction in C/N ratio below 20 shows advanced degree of organic matter stabilization and maturity of organic wastes which makes nutrients easily available to plants^40^. In the present study, azolla and cattledung together brought more decline in C/N ratio of rice straw in comparison to the mixtures having either azolla or cattledung.

Higher phosphorus in all the mixtures of Vcom group in the present study could have been due to phosphorus mineralization and mobilization by the combined action of bacterial and fecal phosphatases of earthworms^41^. Earthworm gut produces alkaline phosphatase, an essential enzyme for biogeochemical cycling of P that facilitates its mineralization^37,42,43^ and converts the state of phosphorus from organic to inorganic, making it available for plants^44^. Increased population of earthworms with time may have enhanced population of phosphate solubilizing microorganisms^45^ and caused more increase in the content of P of all the mixtures of Vcom group. Potassium also showed an upward trend in all the mixtures of present study. RCA of both Vcom and Acom groups showed maximum potassium content but more increase in Vcom could have been due to enzymatic activities of earthworm gut^45^. This could also be due to decomposition of the ingested organic matter in the gut of earthworm and action of endogenic or exogenic enzymes produced by earthworm and microorganisms^37,46^. Earthworm gut harbors a variety of intestinal microorganisms (fungi, bacteria, protozoa and actinomycetes) which produce enzymes such as amylases, proteases, lipases and cellulases enhancing biodegradation of organic matter^22,23^. Azolla is rich in most of the micronutrients^47^, which also seems to be responsible for higher content of potassium in the mixtures of present study. Kaviraj & Sharma^48^, Garg *et al*.^43^ and Suthar^27^, reported an increase in the K content of vermicomposts prepared from various kinds of wastes. However, a decline in K content of vermicompost prepared from coffee pulp was reported by Orozco *et al*.^49^. Highest NPK and other nutrients in the Vcom group suggests potential fertilizer value of the products for crop production^50^ as these are the key elements for plant growth and cell division. The treatment RCA with maximum content of all nutrients shows its potential as a best plant growth medium for sustainable agricultural production^24^. NPK increase growth, vigour and disease resistance of plants and help them to thrive, particularly in changing weather conditions.

 Plants combine hydrogen with carbon during photosynthesis to make carbohydrates and there is a close link in carbon and hydrogen cycles. Hydrogen has been reported to improve seed germination, enzyme activity, oxidative stress tolerance, plant growth and blossom^51–53^. Maximum increase in H in RCA indicates better fertilizer value of this mixture because H maintains cellular homeostasis (via hemeoxygenase-1 enzyme)^52^ involved in signal transduction pathways of plant hormones, can improve resistance of plants to stressors, enhances plant growth (by upregulating phytohormone receptor genes) and delays senescence (by reducing oxidative damage)^54,55^. Increase in hydrogen after vermicomposting/composting in the present study can be due to reduction in carbon and pH of the final product. Lower pH of the products in comparison to initial material has been due to increase in H^+^ concentration during composting^56^. Sulphur is an important component of key enzymes and vitamins necessary for formation of chlorophyll and efficient fixation of nitrogen by plants. It is an essential macronutrient present as sulphate in soils. Plants deficient in S tend to become spindle shaped, stems and petioles become thin, growth is slowed and leaves show chlorosis. S in association with N affects protein and enzyme synthesis of plants. Maximum S in RCA of the Vcom group is expected to increase the yield of plants^57^. Variable increase in sulphur of the products of Vcom in the present study is supported by the findings of Das *et al*.^58^ who related the increase in sulphur after vermicomposting paddy straw, municipal solid waste, cattle manure and weeds by 8–32% due to loss of dry mass as carbon dioxide during mineralization and variable extent of decomposition of different organic wastes. Easha *et al*.^59^ also reported an increase in S after vermicomposting textile mill sludge mixed with cowdung.

An overall decrease was observed in pH of all the treatments but higher decline in the mixtures of Vcom group seems to have helped to conserve nitrogen in the products of Vcom group of present study. Ndegwa & Thompson^60^. Suggested that decrease in pH could be attributed to mineralization of nitrogen into nitrites/nitrates, phosphorus into orthophosphates and bioconversion of the organic material into intermediate species of organic acids. The difference in pH among various treatments of the present study may have been due to a variation in the initial quality of these mixtures which might have affected mineralization process and species of intermediate compounds formed during bioconversion^26^. Shift of pH towards neutral in the final products of all the treatments of the present study can be attributed to mineralization of proteinaceous material that yielded ammonium ions which reduced the level of hydrogen ions during both the processes^41^. Earthworms selectively increase population of catabolically more active microbes^23^, therefore degradation of short chain fatty acids and precipitation of calcium carbonate may have consecutively brought a rise in pH of the products of vermicomposting compared to the products of traditional aerobic composting^28,61^.

In the present study a significant decrease was observed in the EC of all the mixtures after vermicomposting/composting. EC gives the amount of salinity of organic material and is a good indicator to study the quality of vermicompost/compost^62^. In contrast to our findings an increase in EC has been reported after vermicomposting of filter mud due to loss of weight of organic matter and release of available forms of minerals^63^. Similar to our results a decrease in EC of vermicomposted paddy and wheat straw with farmyard manure (1:1 ratio) has been reported due to production of ammonium ions (NH_4_^+^), as well as loss of the dissolved salts in the environment^64^. EC of the products of the present study was in the acceptable range (<0.25 dS/m) for the fertilizers to be used for plants^57^.

The results of present study reveal that RCA and RCFA had maximum sodium which is expected to enhance fertilizer value of the products. Na is considered as a functional plant nutrient^65^ and in the form of oxides, hydroxides and carbonates can increase availability of other nutrients to plants by counteracting soil acidification^30^. The increase could be due to the activity of earthworms as well as due to the nutrient rich azolla as is evident from the initial biochemical composition of azolla. It has been reported that azolla improves the physical and chemical properties of soil by addition of Ca, Mg and Na^66^. Increase in sodium after vermicomposting has also been reported by Bhat *et al*.^67^ but they related it to reduction in the volume of the material after vermicomposting process.

Calcium is an essential plant macronutrient and is required for cell wall stabilization and multiple cell signaling pathways^68^. Maximum content of Ca in the Vcom prepared from rice straw spiked with cattledung and azolla (RCA) would ensure its availability to plants. High content of calcium has been reported in azolla^69^, along with it calciferous glands of earthworm are regarded as excretory organs responsible for eliminating excess calcium from their diet as calcium carbonate^70^. Collectively this might have caused a higher increase in the Ca of Vcom mixtures having azolla. Yan *et al*.^71^ and Spiers *et al*.^72^ reported that calcium oxalate crystals get converted to calcium bicarbonate as the organic waste passes through a worm’s gut. In this form Ca can be easily taken up by plants. Calcium enrichment of the vermicompost due to mineralization and fixation of carbon dioxide to calcium carbonate has been reported by Pattnaik & Reddy^35^. Suthar^25^ has also supported role of earthworm gut for an increase in calcium after vermicomposting sewage sludge mixed with crop residues and cattle dung.

More increase in Mg was observed in the mixtures with straw, azolla, cattledung and fungus in the present study. Magnesium availability from organic matter is affected by the activity of earthworms as fresh casts encourage colonization by fungal and micro-algal hyphae which might be the cause for a higher increase in Mg in the products of Vcom group in the present study^73^. Shak*et al*.^26^ also reported increase in magnesium (17.1–40.8%) after vermicomposting rice residues and related it to the presence of cattle dung which is a rich source of magnesium. However, we have observed that combination of rice straw, cattledung, *A*. *terreus* and azolla is best for increase in magnesium. In contrast to our present findings a decrease of Mg by 3.7–45.7% was reported by Lim *et al*.^74^, after vermicomposting rice husk with *Eudrilus eugeniae*. They suggested that rice residues, which are recalcitrant to degradation due to high lignin content provided unsuitable environment for the growth and livelihood of fungi and microbes. We observed that addition of azolla was responsible for making the environment conducive for *E*. *fetida* and microbes which is evident from the higher contents of nutrients in the mixtures with azolla.

Micronutrients like Fe, Cu, B, Mn, Mo, Zn, Si and Cl are required in a minuscule quantity but exert a greater influence on plant growth as they serve as co-factors of antioxidant enzymes such as superoxide dismutase, catalase and peroxidase etc. Azolla has an ability to concentrate elements like Mn, Cu, Fe, Zn, Ca, Si, N, P from water or element enriched solutions^66^. Thus it could have resulted in a higher increase in the minerals in the mixtures of the present study. Higher micronutrients in the Vcom group in comparison to Acom group of the present study is in accordance with the study of Ghosh *et al*.^75^, who also observed higher content of micronutrients in vermicomposted mixture of cowdung, poultry dropping, kitchen waste and dry leaves in comparison to composted materials. An increase in the micronutrients (Zn by 93.3%, Fe by 77.5%, Mn by 140% and Cu by 166.67%) in vermicompost prepared from different varieties of paddy straw has been reported by Lakshmi *et al*.^41^. They suggested that the variation was due to nature and composition of organic residues and the substrates used. Bansal & Kapoor^39^ observed no change in total P, K and Cu content of compost made from the mixture of crop residues (mustard and sugarcane) and cattle dung and reported that these were being assimilated by the earthworms. However, they noticed significantly more Zn in the compost prepared from cattle dung with earthworms than in the compost without earthworms, similar to the findings of present study. However, Khwairakpam & Bhargava^63^ reported a decrease in Fe and Cu in the vermicompost and related it to their accumulation in earthworm body, release of CO_2_ and water and mineralization processes^76^. Addition of A. *terreus* in the mixtures helped in breakdown of cellulose and lignin making the rigid fibres of paddy softer and more palatable for the action of microbes and earthworms. This is clearly evident from the shortest time (same as RCA) required for bioconversion of RCFA in the present study.

When the best combination RCA (2 parts straw: 1part cattledung: 20 g azolla) was further evaluated by increasing the amount of straw in the mixture (3 and 4 parts of straw: 1 part cattledung: 20 g azolla), the earthworms showed poor growth in the mixtures with 4 parts of straw. However, in the mixture with 3 parts of straw, minor decline was observed in the population buildup and nutrient content (Table 5) along with a slight increase in the time required for degradation (110 days). Therefore, we can opt for bioconversion of this combination of the straw also, as it will decrease the load of such a huge waste left in the fields after harvesting, if quality can be slightly compromised.

## Conclusion

This study clearly indicates that the rice straw, a menace to farmers for storage and usage can be efficiently converted into a nutrient rich plant growth promoting vermicompost with addition of *A*. *terreus*, azolla and cattledung. Vermicomposting brought more increase in N, P, K, H, S, Na, Cu, Zn and Mn with a higher decrease in C/N ratio of all the mixtures. Maximum decline in C/N ratio along with an increase in the quantity of macro and micronutrients and population biuldup of *E*. *fetida* in RCA followed by RCFA make these the best combinations for enhancing agronomic value of rice straw. Our study revealed that 2 parts of rice straw when amended with one part of cattledung and 20 g of azolla provided the best quality vermicompost after 105 days of vermicomposting. At the same time 3 parts of straw could be degraded in a slightly longer time of 110 days and quality of the product was also slightly low. This green technology leads to faster bio-conversion of rice straw into a nutrient-rich organic fertilizer if the residues are mixed with cattledung and azolla. Azolla also reduces the bulk of the mixtures as it reduces dependence on cattledung during composting and vermicomposting.

## Methods

### Procurement of materials and earthworm culture

Young, non clitellate *E*. *fetida* with an average weight (0.50 g) were obtained from the culture beds at the vermifarm of Guru Nanak Dev University, Amritsar and used for vermicomposting. Cattle dung was procured from the dairy farms in the vicinity of the campus. Azolla was procured from a local village pond and cultured in rectangular pits at the University. Each pit was lined with a polythene sheet (10 ft × 10 ft) and the bottom was lined with a 2 cm layer of garden soil and topped with 2 cm cattle dung slurry and filled with water upto 10 cm height. Azolla fronds were released in the pits for multiplication. Pure culture of *A*. *terreus* was procured from the Microbiology Department of GNDU, Amritsar. It was grown and maintained on Potato Dextrose Agar (PDA) and stored at 4 °C till use. Inoculum was prepared in 250 ml Erlenmeyer flasks having 50 ml sterile glucose broth (Glucose + Yeast extract + K_2_HPO_4_ + MgSO_4_ at pH7). Discs with 4 mm diameter were taken from 7 days old culture, added in a flask containing the broth and kept in rotatory shaker at 121 rpm for 42 h at 40 °C before use. Rice straw obtained from the fields on the University campus was dried and chopped (2 mm) before the experiment.

### Experimental design

**Figure 2:**
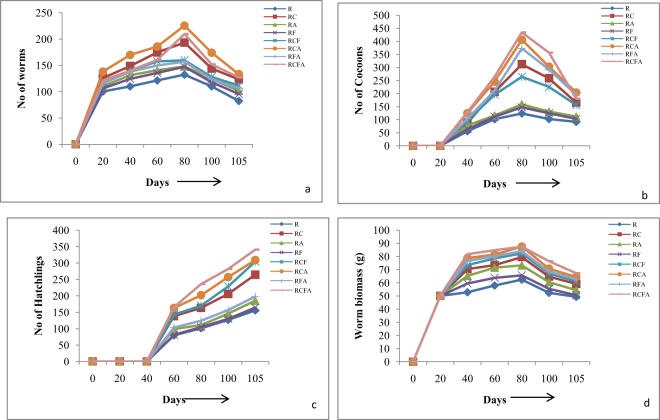
Population buildup of *E*. *fetida* in various mixtures of rice straw, cattledung, azolla and fungus. Values are Mean ± SE. Bars with different superscripts are significant at (p < 0.01).

Experiment was performed in Plastic Tubs (65 × 45 × 30 cm) in triplicate under the sheds. Rice straw (R), Cattle dung (C), Azolla (A) and fungus *A*. *terreus* (F) were mixed in completely randomized design **(**Table 1) to make eight different treatments (1 kg each) viz. rice straw (R), rice straw + cattledung (RC), rice straw + azolla (RA),rice straw + fungus(RF), rice straw + cattledung + fungus (RCF), rice straw + cattledung + Azolla (RCA), rice straw + fungus + azolla(RFA) and rice straw + cattledung + azolla + Fungus (RCFA) and subjected to vermicomposting (Vcom group) and aerobic composting (Acom group). Physico-chemical characteristics of Rice straw (R), Cattle dung (C) and Azolla (A) were estimated before making the mixtures **(**Table 2). The mixtures were kept for 2 weeks for thermal stabilization and removal of volatile toxins prior to inoculation of 100 non-clitellate *E*. *fetida* in each tub of the Vcom group. The mixtures of Acom group were manually turned on alternate days for aeration of the waste. Moisture was maintained by sprinkling water as and when required and by covering the tubs with jute mat. The experiment was terminated when the mixtures were converted to brown earthy material or crumbly balls. Figure 2 shows initial and final appearance of the RCFA mixture of Acom and Vcom groups. Final products were sieved through a nine mesh size sieve, dried in oven for 36 h at 60 °C, packed in poly bags and stored for chemical analysis. The best mixture from this set was selected and the ratio of straw was increased to 3 parts to ascertain the time of degradation and quality of the products (Table 5). This experiment was performed with an aim to reduce the dependence on cattledung.

### Earthworm Biomass and population buildup

Earthworm biomass and population buildup (number of hatchlings, cocoons and number of worms) were recorded in the Vcom group at 20 days intervals till termination of the experiment (0, 20^th^, 40^th^, 60^th^, 80^th^, 100^th^ and 105^th^ day post inoculation). Worms, cocoons and hatchlings were sorted, their numbers were recorded separately for each treatment and put back in the tray immediately.

### Physico-chemical analysis

Physico-Chemical analysis of the mixtures was carried out separately. Decibel soil and water analyzer kit was used for estimation of pH and EC from the distilled water suspension of each mixture (1:10 w/v). Total organic carbon, nitrogen, hydrogen and sulphur were analyzed with CHNSO analyzer, Thermo Flash-2000. Powdered sample (1–3 mg) was taken in a capsule and combusted at 1000 °C with helium as a carrier gas and oxygen for combustion. Eager experience software was used for elemental analysis. Nutrients (P, K, Ca, Na, Mg, Bo, Cu, Fe, Zn and Mn) were analyzed with Thermo Scientific iCAP 6000 series ICP spectrometer. Sample (0.1 g) was digested in a diacid mixture (HCl + HNO_3_ 1 ml: 5 ml) in Anton Parr Microwave Multiwave 3000 for 75 minutes. After digestion the material was diluted to 50 ml with double distilled water, filtered and subjected to iCAP analysis.

### Statistical analysis

One way ANOVA and Tukey’s test were used for calculating the variation and significance level (P < 0.01) between means of different treatments with the help of SPSS 16 program.

## Data Availability

The datasets that support the findings of this study are available with the corresponding author.
